# Is There an Association between the Use of Social Media and Self-Rated Health?

**DOI:** 10.3390/bs13090777

**Published:** 2023-09-18

**Authors:** Kholisani Ndlovu, Lebogang M. Ramalepe, Nwamaka C. Nwogwugwu, Bukola G. Olutola

**Affiliations:** 1 School of Engineering, Science and Health, The Independent Institute of Education (IIEMSA), 144 Peter Rd, Ruimsig, Roodepoort 1724, Gauteng, South Africa; st10318087@advtechonline.mail.onmicrosoft.com (K.N.); nnwogwugwu@iiemsa.co.za (N.C.N.); 2 School of Social Science, The Independent Institute of Education (IIEMSA), 144 Peter Rd, Ruimsig, Roodepoort 1724, Gauteng, South Africa; lramalepe@iiemsa.co.za

**Keywords:** self-rated health, social media, education, marriage, employment

## Abstract

This study sought to determine the association between social media and self-rated health. This study used the 2022 Health Information National Trends Survey for American adults. A statistical analysis was conducted using Chi-square and multivariable logistic regression. Of the 6018 study participants, the majority reported that they were in excellent/very good and good health (82.9%, *n* = 4930). More than half (58.2%, *n* = 3268) of the respondents reported that they visited a social media site almost every day in the past 12 months, while 76.8% (*n* = 4843) reported that they never interacted with people who had similar health or medical issues on social media. There was no association between everyday visits to a social media site, interaction with people with similar health/medical issues on social media, or watching a health-related video on social media and self-rated health. Those who had full-time employment were more likely to rate their health as excellent/very good/good (AOR: 2.394, 95% Conf. Int: 1.820–3.149) compared to those with no full-time employment. Marital status, confidence in taking care of oneself, education, and current smoking were associated with self-rated health. This study showed that the use of social media which included the watching of health-related videos was not associated with self-rated health.

## 1. Introduction

Social media is a digital system that allows users to create, share, and trade user-generated content like messages, photos, videos, and audio recordings through virtual networks, such as Facebook, Twitter, Instagram, etc. Worldwide, 2.9 billion people use Facebook as of January 2023, with India having the highest number of users, followed by the United States, Indonesia, and Brazil [1]. Furthermore, a study conducted in 2021 showed that in the United States, younger Americans aged 18–24 years use a variety of social media platforms frequently (2). Among this age group, 78% use Snapchat, 71% use Instagram, and 45% use Twitter, and the majority of adults (68%), with the exception of older adults aged 65 years and above, use Facebook [2].

Over the last decade, social media has profoundly changed the sphere of interpersonal communication, with 60% of the world’s population using social media, and the United States of America (USA) being the third largest user of social media platforms after India and China, with 90% of Americans using social media [3]. Social media’s appeal can be attributed in part to the accessibility of public message creation and distribution at a minimal cost [4].

With the ability to spread information quickly and widely, social media has been used as a tool for health objectives by providing unrestricted access, sharing and content creation of health promotion and education content, medical services, and administration [5]. This has led to a significant influence on people’s mental and physical health; therefore, it is impossible to overlook how social media affects health [6].

Social media has been integrated into health intervention to promote positive actions like physical activity and interpersonal connection, providing platforms for global peer support [5,6]. For instance, in Qatar, a weight loss campaign was successful due to social media, which was used as a tool for health behavior change motivation, progress sharing, as well as group goal achievement sharing [7]. Although social media offers numerous benefits for general health, there are also evident drawbacks such as exposure to unhealthy behaviors like tobacco use, alcohol abuse, and other behavioral issues [8].

A study conducted by Bakalu, McCloud, and Viswanath (2019) examining the association between social media use with social wellbeing, positive mental health, and self-rated health found that age, education, income, race, and ethnicity were significant predictors of self-rated health [6]. Self-rated health has been shown to be consistent with objective health status and has been used as a global measure of health status in the general population [9,10]. Additionally, various studies have shown that self-rated health is associated with mortality and morbidity, as well as disability [10]. Other associated health predictors and outcomes include physical health status, mental health status, and functional limitations [11].

The effect of social media on self-rated health is a complex issue that needs further investigation. This is particularly important given how prevalent social media platforms are in daily life and how many individuals use them for communication and self-expression. Many studies have looked at the association between general social media use and self-rated health [6,12]. However, none have looked at specific the use of social media for health reasons and its effects on self-rated health. In this study, the use of social media to share general health-related information, interact with people with similar health or medical issues on social media or online forums, and watch health-related videos on social media were used as measures of social media. Therefore, this study explored the association between these measures of social media and self-rated health.

## 2. Materials and Methods

Data for this cross-sectional study were obtained from the Health Information National Trends Survey 6 (HINTS 6) which was conducted from 7 March 2022 to 8 November 2022. The sampling frame for HINTS 6 consisted of a database of addresses used by Marketing Systems Group (MSG) to provide random samples of addresses. All non-vacant residential addresses in the United States present on the MSG database, including post office (P.O.) boxes, throwbacks (i.e., street addresses for which mail is redirected by the United States Postal Service to a specified P.O. box), and seasonal addresses, were subject to sampling. The sampling frame of addresses was grouped into four explicit sampling strata,

1. Addresses in urban areas with high concentrations of minority adults (HM urban);
2. Addresses in urban areas with low concentrations of minority adults (LM urban);
3. Addresses in rural areas with high concentrations of minority adults (HM rural); and
4. Addresses in rural areas with low concentrations of minority adults (LM rural).

The sampling strategy for the HINTS 6 survey consisted of a two-stage design. In the first stage, a stratified sample of addresses was selected from a file of residential addresses. In the second stage, one adult was selected within each sampled household. Of the 6505 questionnaires received, 27 were returned blank, 148 were determined to be incompletely filled out, and 78 surveys were identified as duplicates (i.e., the same household returned multiple surveys). The remaining 6252 surveys were determined to be eligible. The full methodology can be found on the HINTS website [13].

### 2.1. Measures

#### 2.1.1. Self-Rated Health

Self-rated health was measured by the question “In general, would you say your health is….?” Options “Excellent”, “Very good” and “good” were categorized as good while “fair” and “poor” were categorized as “poor”.

#### 2.1.2. Behavioral Measures

##### Use of Social Media

Social media was measured by asking if the respondents visited a social media site, shared general health-related information on social media, interacted with people who had similar health or medical issues on social media or online forums, and if they watched a health-related video on a social media site, all in the past 12 months. The responses to all the questions were “Almost every day”, “At least once a week”, “A few times a month”, “Less than once a month”, and “Never”. The respondents were asked about their level of confidence in finding helpful health resources on the internet. Those who said that they were completely confident or very confident or somewhat confident were categorized as confident, while those who said that they were a little confident or not confident at all were classified as not confident for the purpose of the analysis.

##### Use of Apps and Wearables

The respondents were asked if they had used a health or wellness app on their tablet or smartphone in the past 12 months. They were also asked if they had used an electronic wearable device or tracked their health or activity in the past 12 months. The responses were Yes/No.

Current smoking was measured by asking the participants how often they smoked with options “everyday”, “some days”, and “not at all”. Respondents were asked how confident they were about their ability to take good care of their health (confidence in self-health care). The options were “Not confident”, “a little confident”, “somewhat confident”, “Very confident”, “Completely confident”.

##### Socio-Demographic Measures

Age, birth gender, education, employment status, and marital status of the respondents were asked. For education, respondents were asked about the highest grade or schooling they completed. For the statistical analysis, they were classified into Grades 1–11, Grade 12, and higher than a grade 12 level of education. To measure employment status, they were asked if they usually worked 35 h or more per week in total at all jobs or businesses in the past 30 days. For the purpose of analysis, those who said “yes” were classified as those who worked 35 h or more per week in the past 30 days while those who said “no” were classified as those who worked less than 35 h per week. Marital status was classified into married, living as married, or living with a romantic partner as one category, divorced, widowed, and separated respondents were classified as another category, while those who were single and never married are one category.

### 2.2. Data Analysis

To account for selection probabilities and the complex sample design used in the HINTS 6, weight adjustments were made and the data analysis was performed in a survey mode using the “svy” command in STATA version 12 (Stata Corporation, College Station, TX, USA). Descriptive analyses were carried out to explore the prevalence of self-rated health and the differences between the groups were tested using Chi-square tests. Multivariable logistic regression was performed to determine the independent association between self-rated health and social media after controlling for potential confounders such as participants’ socio-economic status, current smoking, and other variables. The criterion for inclusion of the variables into the multivariable logistic model from the bivariate analysis (Chi-Square test) was set at *p* < 0.05. Statistical significance was also set at *p* < 0.05 for the regression models.

## 3. Results

Out of the 6252 respondents who were eligible in the HINTS 6 survey, 6018 respondents answered the question on self-rated health. More than eighty percent of them (82.9%; *n* = 4930) reported that they had excellent or very good or good health. A little over half of the respondents were females (50.8%; *n* = 3535) and between the ages 18 and 49 years (51.2%; *n* = 2179). Almost three quarters of them (71.5%; *n* = 4393) had more than a high school education. Regarding the use of social media, 58.2% (*n* = 3268) of the respondents visited a social media site in the 12 months preceding the survey, while only 16.2% (*n* = 1271) never visited any social media site. Conversely, only 1.5% (*n* = 77) of the respondents reported that they interacted almost every day with people who had similar health or medical issues in social media or online forums in the 12 months preceding the survey (Table 1).

### 3.1. Prevalence of Self-Rated Health Based on the Socio-Demographic and Behavioral Factors

In Table 2, no statistical significance was found between males and females with regard to self-rated health. Also, there was no difference in the prevalence of good self-rated health with regard to age. A higher proportion of those who were married or lived as romantic partners (86%; *n* = 2574) rated their health as excellent or very good or good than those who were single, never married (80.1%; *n* = 888), and those who were divorced or widowed or separated (75.6%; *n* = 1283) (*p*-value=0.001). More respondents who worked for 35 h or more in a week rated their health as excellent/very good/good than those who worked for less than 35 h/week (88.9% vs. 75.5%; *p*-value < 0.001).

For smoking status, the prevalence of self-rated health was highest among non-current smokers (84.8%) than those who smoked some days (68.4%) and those who smoked everyday (69.8%) at the time of the survey (*p* = 0.003). Similarly, the prevalence of excellent/very good/good self-rated health was higher among those who reported that they noticed calorie information listed next to the food on their menu at a fast food or sit-down restaurant than those who did not (85.3% vs. 80.5%, *p* = 0.008).

### 3.2. Prevalence of Excellent/Very Good/Good Self-Rated Health among Social Media Users

About 85% of the respondents who were very confident/confident in finding helpful health resources on the internet rated their health as excellent/very good/good. This was more than the prevalence of excellent/very good/good self-rated health among those who had a little confidence or no confidence in finding helpful health resources on the internet (69.2%) (*p* < 0.001). No significant difference was observed in the prevalence of self-rated health among the different frequencies of visits to a social media site in the 12 months preceding the survey. However, those who watched health-related videos on social media site almost everyday in the 12 months preceding the survey had the lowest prevalence of excellent/very good/good self-rated health (67%), while those who watched a health-related video on a social media site for less than a month in the 12 months preceding the survey had the highest prevalence (85.5%; *n* = 2058) (Table 3).

### 3.3. Associated Factors of Excellent/Very Good/Good Self-Rated Health

In the multivariable logistic regression, none of the social media variables were associated with self-rated health. The study respondents who worked for 35 h/week or more were more likely to rate their health as excellent/very good/good compared to those who worked for less than 35 h/week (AOR: 2.40: 95% Conf. Int: 1.82–3.15). The study respondents who were divorced or widowed or separated were less likely to have excellent/very good/good self-rated health compared to those who were married or living with a romantic partner (AOR: 0.62: 95% Conf. Int: 0.46–0.83). However, there was no significant difference between the married or those living with a romantic partner and those who were single and never married (Table 4).

## 4. Discussion

Our findings suggest that several factors influence self-rated health; however, social media was not one of them. Although the usage of social media sites was high, our results indicated that there were no associations between interacting with people of similar health or medical issues on social media, visiting a social media site in the past 12 months, sharing of general health-related information on social media, watching of health-related videos on social media in the 12 months preceding the survey, and self-rated health. Employment, having confidence in taking care of one’s health, smoking status, marital status, and education were the factors associated with self-rated health.

In the current study, more than 80% of the respondents visited a social media site daily to less than once a month within the last 12 months of the survey. This is in a way like what was reported by Pew Research (2021) [2], which showed that seven in ten American citizens reported that they ever used social media. Most of the current study’s respondents either watched health-related videos on social media for less than a month or never watched at all. More than a quarter never used social media as a tool for interacting with people with similar health conditions or watching health-related videos. This is particularly interesting as a previous study suggested the importance of the use of social media as a tool for connecting with people with similar health-related illnesses [6,14,15], especially post COVID-19 [16]. In addition, the benefits of watching a health-related video have been linked to health promotion [17,18], prevention [19,20], and management [21]. None of the measures of social media use in this study were associated with self-rated health. However, many studies have shown an association between the use of social media and self-rated health [6].

Social media provides social support and can help people who do not have physical support groups [22], as those who feel less connected to their family are more likely to rely more on social media for social interactions and connectedness [23]. However, Lewandowski et al., 2011, showed that individuals who indicated that the bulk of their social support came via face to-face communication reported feeling significantly better following a supportive interaction when compared with those who reported receiving support via telephone or the Internet [24].

Furthermore, in the current study, divorced/widowed/separated individuals were less likely to rate their health as excellent/very good/good compared to those who were married or living with a romantic partner. Marriage can be seen as a form of social support, and this also confirms that face-to-face support is important when it comes to one’s perception about one’s health. Many studies have shown that marriage or living with a partner is associated with good self-rated health [25,26]. Conversely, the stress of union dissolution or loss of partner may leave previously married individuals in poorer health, relative to married people [27], making them not rate their health as good. Divorce or separation can exert a significant impact on the structure of a person’s social network, making those who divorce become less popular in their social circle [28], giving rise to lack of face-to-face support or connection.

Also, widowhood can lead to low levels of bonding trust, which may eventually lead to poor self-rated health [29]. Social psychology literature indicates that the loss or lack of a significant other is associated with poor self-rated health [30,31,32]. However, the current study did not show any significant difference between self-rated health among the married and those who were single but never married. This is contrary to the findings of many studies that showed that being single is associated with having poor self-rated health [25].

We found strong evidence that confidence in taking care of oneself is associated with excellent self-rated health. This is unsurprising as several studies have reported similar findings [33,34,35,36]. Confidence in taking care of oneself is positively correlated to good/excellent self-rated health. The more confidence someone has in taking care of himself, the more the likelihood of having good self-rated health.

The results from this study suggest that full-time employment (employed for more than 35 h a week) is linked to excellent self-rated health. This is in line with several studies that have found that full-time employment is associated with high self-rated health, for example, Krokavcova et al. (2010) [37] conducted a study on patients with multiple sclerosis. Their findings suggested that employed patients with multiple sclerosis were more likely to report good self-rated health than those with part-time employment. In a recent study, An and Park (2022) [38] concluded a longitudinal study on precarious employment and self-rated health among young adults in Korea. Similarly to the findings in the current study, precarious (part-time) employment was less associated with good self-rated health compared to full time employment.

Participants who reported 12 or more years of education were more likely to have good/excellent self-rated health compared to those with less than 11 years of education. Numerous studies concur with our findings. Schellekens and Ziv (2020) analyzed the trends in education and self-rated health in the United States from 1972 to 2018. Self-rated health improved with the increase in educational attainment [39].

Participants who smoked regularly or occasionally were less likely to report excellent self-rated health than those who never smoked. This has particularly contributed to the awareness and knowledge around the health effects and hazards of using tobacco products [40,41]. Over the past two decades, more people have become conscientized and sensitized to the dangers of tobacco [42]. Thus, people who smoke are less likely to report good self-rated health.

The results of this study need to be interpretated within the limitations of the study design. This was a cross-sectional study; therefore, no causal association can be established as well as no temporal order of event. The study cannot establish that the factors shown to be associated in this study are the causes of poor or bad self-rated health. The study also relied on self-reported information, which might lead to misclassification bias. Also, the data used for the study did not have all the confounders that were used in previous studies to look at the relationship between self-rated health and social media; however, with the data, we were able to establish that there was no association between the social media measures and self-rated health. The dichotomization of the outcome variable self-rated health might have increased a Type 1 error in the study [43]. Another limitation of the study is that the study was conducted during the COVID-19 period and the pandemic might have had considerable implications for individual and collective health, as well as for emotional and social functioning. This might have a negative effect on how the participants rated their health [44]. A major strength of this study lies in the use of a large, nationally representative sample of all Americans to explore the association between self-rated health and social media.

## 5. Conclusions

The current study investigated the association between self-rated health and the use of social media. However, this study did not find any association between the two. The study showed that social media did not have an effect on how people feel, even when they watched health-related videos on social media. Socio-demographic factors such as employment, education, and marital status were found to be important factors of self-rated health. Others include tobacco smoking and confidence in taking care of oneself.

## Figures and Tables

**Table 1:** Characteristics and demographics of the respondents.

Characteristics		% (*n*)
Self-rated health		
	Excellent/very good/good	82.9 (4930)
	Fair/Poor	12.1 (1088)
Age of study respondents		
	18–34	25.9 (939)
	35–49	25.3 (1240)
	50–64	27.3 (1772)
	65–74	13.0 (1356)
	75 years and older	8.6 (847)
Age of people in the household	18 years and older in the household	80.2 (4116)
	<18 years in the household	19.8 (2136)
Gender		
	Male	49.2 (2307)
	Female	50.8 (3535)
Education		
	1–11 years of education	6.9 (387)
	12 years of education	21.6 (1068)
	>12 years of education	71.5 (4393)
Employment		
	<35 h/week employment	45.4 (3062)
	≥35 h/week employment	54.6 (2778)
Marital status		
	Married/living as married or living with a romantic partner	55.9 (2997)
	Divorced/Widowed/Separated	12.9 (1721)
	Single, never married	31.2 (1119)
Confidence in self-health care		
	Not confident	1.6 (78)
	Somewhat confident/ a little confident	29.7 (1657)
	Completely confident/very confident	68.7 (4301)
Current smoking		
	Not at all	88.0 (5228)
	Someday	4.1 (195)
	Everyday	7.8 (449)
Notice of calories in food menu		
	No	51.1 (3051)
	Yes	48.9 (2903)
Confidence in finding helpful health resources on the internet.		
	A little confident/Not confident at all	13.3 (957)
	Completely/very/somewhat confident	86.7 (5163)
Health wellness app usage		
	No	33.0 (1852)
	Yes	56.6 (3044)
	No health apps on the phone or tablet	10.5 (622)
Wearable device usage		
	No	63.3 (4168)
	Yes	36.7 (2074)
Socia media site visit		
	Almost everyday	58.2 (3268)
	At least once a week	11.8 (753)
	A few times a month	8.3 (481)
	Less than a month	5.4 (387)
	Never	16.2 (1271)
Sharing of health information on social media		
	Almost everyday	1.2 (59)
	At least once a week	2.2 (134)
	A few times a month	7.8 (402)
	Less than a month	20.0 (1238)
	Never	69.0 (4305)
Online health interaction		
	Almost everyday	1.5 (77)
	At least once a week	2.6 (136)
	A few times a month	6.4 (301)
	Less than a month	12.7 (781)
	Never	76.8 (4843)
Watching of health-related videos on social media		
	Almost everyday	3.3 (171)
	At least once a week	7.3 (419)
	A few times a month	18.9 (1047)
	Less than a month	30.1 (1836)
	Never	40.4 (2685)

**Table 2:** Prevalence of excellent/very good/good self-rated health with regard to socio-demographic and behavioral factors.

Characteristics		% (*n*)	*p*-Value
Age of study participants			0.134
	18–34	85.0 (769)	
	35–49	84.2 (988)	
	50–64	83.0 (1393)	
	65–74	81.4 (1076)	
	75 years and above	75.8 (641)	
Age	18 years and older in the household	83.7 (3323)	0.055
	<18 years in the household	79.8 (1607)	
Gender			0.685
	Male	83.2 (1911)	
	Female	82.5 (2835)	
Education			<0.001
	1–11 years of education	62.5 (230)	
	12 years of education	79.4 (802)	
	>12 years of education	86.3 (3719)	
Employment			<0.001
	<35 h/week employment	75.5 (2292)	
	≥35 h/week employment	88.9 (2452)	
Marital status			0.001
	Married/living as married or living with a romantic partner	86.0 (2574)	
	Divorced/Widowed/Separated	75.6 (1283)	
	Single, never married	80.1 (888)	
Confidence in self-health care			<0.001
	Not confident	27.7 (17)	
	Somewhat confident/ a little confident	63.1 (967)	
	Completely confident/very confident	92.8 (3935)	
Current smoking			0.003
	Not at all	84.8 (4340)	
	Someday	68.4 (135)	
	Everyday	69.8 (297)	
Notice of food calories in food menu			0.008
	No	80.5 (2368)	
	Yes	85.3 (2469)	

**Table 3:** Prevalence of excellent/very good/good self-rated health among social media users.

Characteristics		% (*n*)	*p*-Value
Confidence in finding helpful health resources on the internet.			<0.001
	A little confident/Not confident at all	69.2 (638)	
	Completely/very/somewhat confident	85.2 (4206)	
Health wellness app usage			0.0261
	No	82.4 (1434)	
	Yes	85.5 (2536)	
	No health apps on the phone or tablet	78.2 (475)	
Electronic wearable device usage			0.0363
	No	81.1 (3186)	
	Yes	86.0 (1739)	
Social media site visit			0.044
	Almost everyday	84.5 (2651)	
	At least once a week	87.3 (618)	
	A few times a month	79.3 (380)	
	Less than a month	72.9 (291)	
	Never	79.3 (941)	
Sharing of health information on social media			0.232
	Almost everyday	88.6 (47)	
	At least once a week	79.0 (96)	
	A few times a month	76.9 (318)	
	Less than a month	86.3 (1016)	
	Never	82.8 (3394)	
Online health interaction			0.092
	Almost everyday	83.6 (56)	
	At least once a week	71.4 (91)	
	A few times a month	72.6 (229)	
	Less than a month	84.2 (606)	
	Never	84.0 (3887)	
Watching of health-related videos on social media.			0.017
	Almost everyday	67.0 (124)	
	At least once a week	85.6 (334)	
	A few times a month	82.3 (843)	
	Less than a month	86.5 (1523)	
	Never	81.4 (2058)	

**Table 4:** Factors associated with excellent/very good/good self-rated health.

Characteristics		Adjusted Odds Ratio (95% Confidence Interval)	*p*-Value
Employment			
	<35 h/week employment	1.0	
	≥35 h/week employment	2.40 (1.82–3.15)	<0.001
Confidence in self-health care			
	Not confident	1.0	
	Somewhat/a little confident	4.06 (1.65–9.96)	0.003
	Completely confident/very confident	34.8 (14.9–80.9)	<0.001
Current smoking			
	Everyday	1.0	
	Someday	1.64 (0.60–4.48)	0.330
	Not at all	1.89 (1.24–2.89)	0.004
Marital status			
	Married/living as married or living with a romantic partner	1.0	
	Divorced/Widowed/Separated	0.62 (0.46–0.83)	0.002
	Single, never married	0.86 (0.63–1.18)	0.345
Education			
	1–11 years of education	1.0	
	12 years of education	2.19 (1.39–3.45)	0.001
	>12 years of education	2.85 (1.85–4.38)	<0.001

## Data Availability

These are publicly available data.
